# Anatomical Basis for Preservation of Cartilaginous Apophysis in Pediatric Patients Undergoing Iliac Crest Bone Graft Harvest: A Cohort Study

**DOI:** 10.7759/cureus.58020

**Published:** 2024-04-11

**Authors:** Sneha Pendem, Kathiravan Selvarasu, Murugesan Krishnan, Santhosh P Kumar, Raparthi Bhuvan Chandra

**Affiliations:** 1 Oral and Maxillofacial Surgery, Saveetha Dental College and Hospitals, Saveetha Institute of Medical and Technical Sciences, Saveetha University, Chennai, IND

**Keywords:** pediatrics, innovative surgical techniques development, novel technique, alveolar bone grafting, anterior and posterior iliac spine, iliac bone

## Abstract

Background

The anterior iliac crest is the workhorse for the harvest of cancellous bone in children undergoing cleft alveolar bone grafting. However, the complexity of the anatomy makes the process of harvesting graft technique sensitive. The aim was to describe the outcomes of the medially based trap door method of graft harvest in pediatric patients undergoing cleft alveolar bone grafting.

Methods

A cohort study was conducted, including all the patients in the age group of 8-12 years. Alveolar bone grafting was performed after harvesting a cancellous graft from the iliac crest bone grafting (ICBG) using a medially based trap door approach. Intraoperative time, average blood loss, and postoperative outcomes, including pain score, paresthesia, and gait disturbances, were recorded.

Results

A total of 28 patients were included in the study based on the inclusion and exclusion criteria. The volume of cancellous bone harvested was between 4-9 cc. The mean intraoperative time was 42 minutes, with an average blood loss of 36 to 48 ml. The average visual analog scale (VAS) score in the postoperative period was 3.5, 6, and 4 on the first, third, and seventh postoperative days. All the patients were ambulated on the second postoperative day, and none of them reported paresthesia. Long-term evaluation of the anterior illum revealed intact crestal morphology with a bone refill on the posterior-anterior (PA) pelvic X-ray.

Conclusion

A medially based modified trap door approach is more efficacious and less morbid for the harvest of ICBG in pediatric patients.

## Introduction

The anterior iliac wing is regarded as the workhorse for maxillofacial osseous reconstruction in pediatric patients. It is an excellent source of cortical, cancellous, and cortical cancellous bone grafts, which can promote bone healing at the recipient site via osseo-conduction, induction, and osteogenic properties [1]. Several graft harvesting techniques have previously been proposed to facilitate graft harvest from the adult anterior iliac wing. These techniques range from using minimally invasive trephines and splitting the crestal ridge to harvesting the tricortical strut. However, each of these techniques has its own set of complications, which vary in intensity of presentation. While postoperative pain (72% in the immediate postoperative phase to 22% after one month post-op), gait disturbances (8.9% to 27.7% at the end of three months), and paresthetica meralgia due to injury to the lateral cutaneous nerve of the thigh (8.3%) are the most common, adynamic ileus and abdominal content herniation have also been reported infrequently [2,3]. One of the most uncommon issues reported with iliac crest bone grafting (ICBG) harvesting is hemipelvic growth discrepancies in pediatric patients caused by iliac apophysis injury [4].

The primary role of the cartilaginous apophysis at the crest is to facilitate pelvic growth [4]. Injury to this cartilage has been shown to disrupt growth in the hemipelvis [4-6]. It is therefore essential to maintain the integrity of the iliac apophysis without compromising its vascular supply during the graft harvesting process. Except for the laterally based trap door approach, traditional cancellous bone harvesting techniques from the anterior iliac crest eventually cause cartilaginous apophysis damage. The lateral-based trap door approach necessitates the removal of lateral muscular attachments in order to maintain the subperiosteal plane, which can cause healing to take longer. To address this issue, we proposed a medially based trap door approach that uses the abdominal obliques and iliacus muscle to gain access to the cancellous bone and medial cortex in pediatric patients undergoing cleft alveolar bone grafting.

The purpose of this paper was to describe the results of a methodically based trap door approach for harvesting cancellous bone in pediatric children undergoing cleft alveolar bone grafting.

## Materials and methods

Study design

A retrospective cohort study was conducted with ethical approval number IHEC/SDC/FACULTY/21/OMFS/011, enrolling all the pediatric patients within the age group of 8-12 years who reported to our center for the management of unilateral or bilateral cleft alveolar defects. The preoperative evaluation included a clinical and radiographic examination of the defect, as well as an orthodontic opinion on transverse maxillary expansion. The study included patients who underwent alveolar bone grafting after transverse maxillary expansion and post-alveolar fistula closure, as well as when the root of the tooth in the line of the eruption was two-thirds completed. The study excluded patients undergoing revision alveolar bone grafting who had previously undergone graft harvest from the anterior iliac bone, as well as adolescents undergoing ICBG harvest.

The volume of the cleft alveolar defect site was evaluated prior to surgery using maxillary computed cone beam tomography (CBCT). The CBCT records (16 cm x 17 cm FOV, 120 kV, 5 mA, 24 s) were analyzed using the CS imaging software (Carestream Dental LLC, Atlanta, United States) with a slice thickness of 3 mm. Only scan volumes with the highest quality were included. The right hip was chosen as the harvest site for the graft. To harvest cancellous bone grafts, all patients used a modified medially based trap door technique with a lateral cutaneous approach for the anterior iliac bone.

Surgical technique

**Figure 1:**
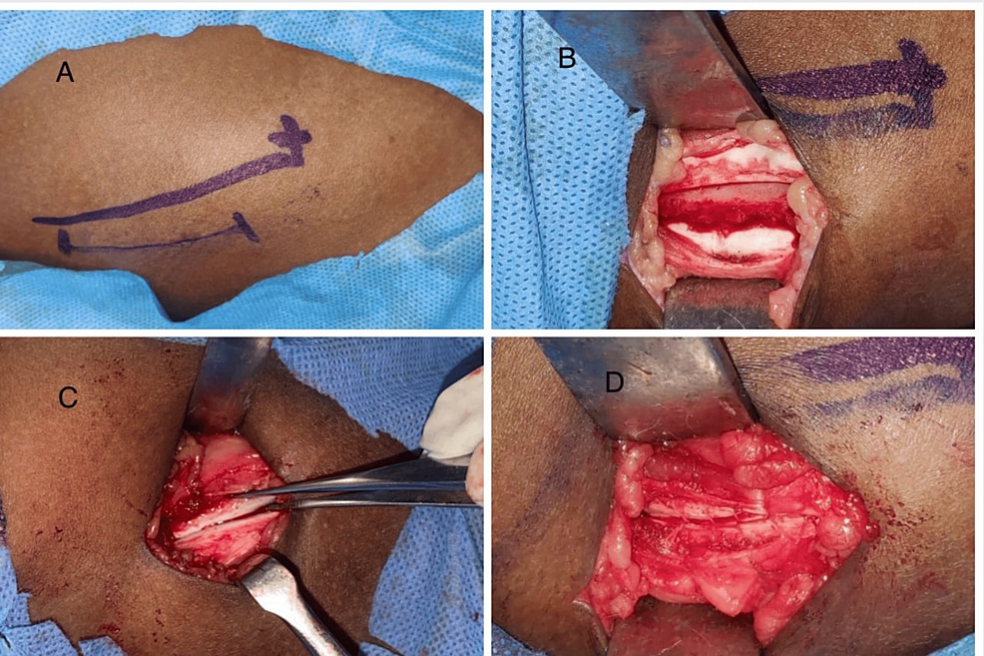
(A) Incision of 3-4 cm placed 1 cm lateral to the crest and 1 cm superior to the ASIS; (B) Medially based trap door to access the cancellous bone of the anterior iliac crest; (C) Suturing the cartilaginous cap to the lateral periosteum without compromising apophyseal anatomy; (D) Layer-wise closure ASIS: Anterior superior iliac spine

The current technique involves approaching the anterior iliac crest through a lateral cutaneous incision. After administration of local anesthetic lidocaine with 1:80,000 adrenaline, an incision of 3-4 cm was placed 1 cm lateral to the crest and 1 cm superior to the anterior superior iliac spine (ASIS) (Figure 1A). The length of the incision can be extended based on the requirement of being 1 cm short of the anterior superior iliac spine inferiority and the tuberosity superiorly. Dissection was done to expose the avascular aponeurosis of the external oblique abdominis and the tensor fascia.

The fascia was incised lateral to the crest to access the cartilaginous apophysis. Anterior and posterior stop cuts (incision over the cartilage 3-scar away) were placed over the crestal cartilage, leaving the medial cartilaginous wall intact, and these were connected by incising the cartilage lateral to the iliac crest (lateral junction of the cartilage with the lateral cortex). The medial wall of the cartilage was kept intact to maintain vascular supply to the cartilaginous trap door, creating a medial-based trap door to access the cancellous bone of the anterior iliac crest (Figure 1B). This trap door was pedicled on the abdominal obliques and the iliacus, thus maintaining the vascular integrity of the cartilaginous apophysis. A subperiosteal tunnel can be created on the medial side to harvest the medial cortical plate if necessary. Once the graft harvest was complete, hemostasis was achieved by the application of bone wax, followed by suturing the cartilaginous cap to the lateral periosteum without compromising (Figure 1C) the apophyseal anatomy, after which the wound was sutured in layers (Figure 1D).

An indwelling catheter was placed transcutaneously to facilitate the injection of a long-acting local anesthetic for postoperative pain control, which was removed on the third postoperative day. The standard postoperative antibiotic regime of cephalosporins was followed to avoid surgical site infection.

Evaluation

**Figure 2:**
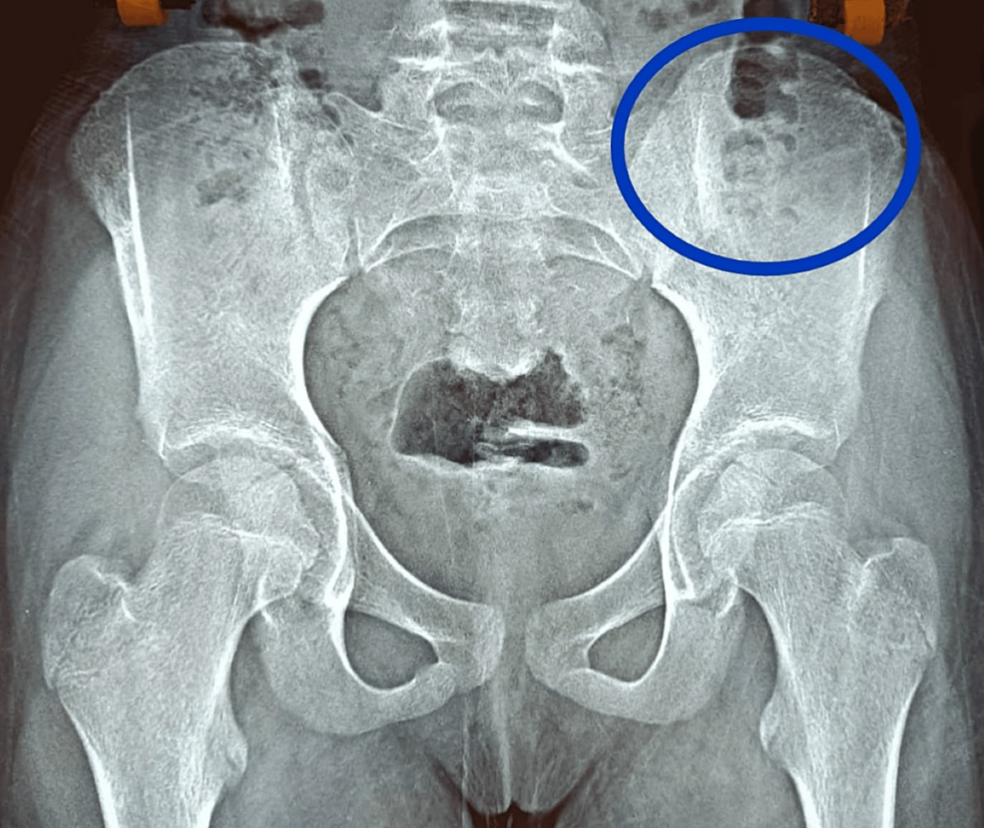
Postoperative radiograph after six months showing the integrity of iliac apophysis

Intraoperatively graft harvest time (from incision to closure) and blood loss were recorded. Postoperative pain (visual analog scale (VAS) score: POD 1, 3, 5), ambulation time, gait disturbances (POD 5), and paresthesia along the lateral thigh were assessed to assess the efficacy of the technique. A postoperative posterior-anterior (PA) view of the pelvis was recorded after six months of harvest for all patients at the end of six months to assess the integrity of the iliac apophysis, as shown in Figure 2.

## Results

A total of 32 patients with unilateral or bilateral cleft alveolus requiring alveolar bone grafting were enrolled in the study. Four patients were excluded based on the exclusion criteria. All the patients underwent the preoperative foresaid evaluation. The mean age of the patients undergoing alveolar bone grafting was 9-12 years. Five patients were grafted for bilateral cleft alveolus, while the rest of the 23 patients had unilateral cleft alveolus. The average volume of bone graft needed was 3.9 to 9 cm^2^. The medial cortical plate (12-15 mm) was harvested to support the nasal floor.

**Figure 3:**
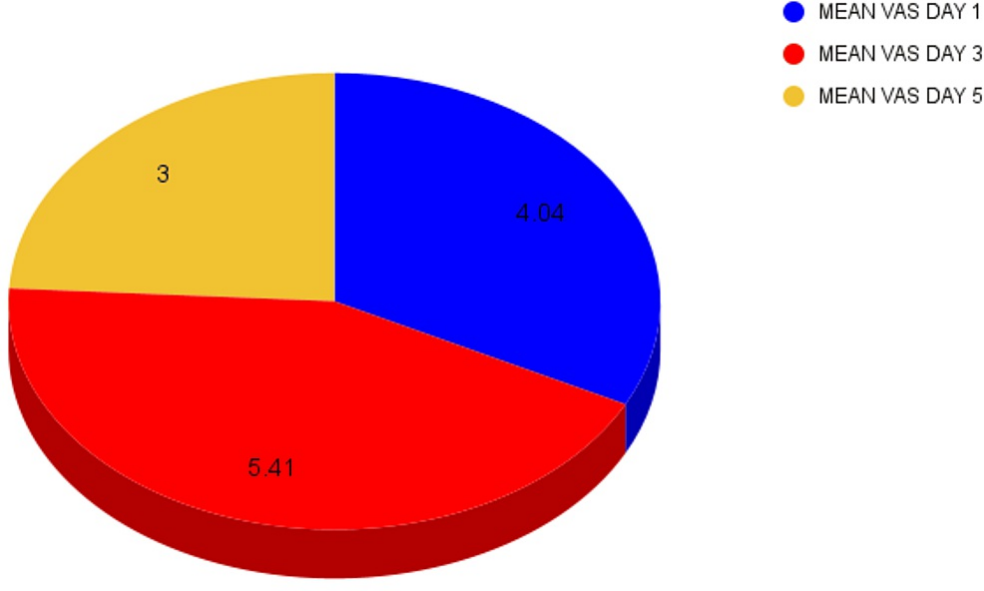
Pie chart depicting the mean VAS score on days 1, 3, and 5 VAS: Visual analog scale

The mean time for the harvest of graft was 43 minutes (42 to 50 minutes). The average volume of blood loss ranged between 36 and 48 ml (4-5 gauze used, 10 x 10 cm), which was determined using the gauze visual analogue method. The mean VAS score for postoperative pain was 3.5 (2-5) on the first postoperative day, while it was between 4-7 (mean VAS 6) on the third postoperative day and reduced to 3-5 by the fifth postoperative day, as explained in Table 1 and Figure 3.

**Table 1:** Mean VAS scores on postoperative days 1, 3, and 5 VAS: Visual analog scale; *: Statistically significant

	Mean	Standard Deviation	p-value
VAS on POD 1	4.04 ± 0.173	0.898	0.00*
VAS on POD 3	5.41 ± 0.187	0.971	0.00*
VAS on POD 5	3.00 ± 0.151	0.784	0.00*

All the patients were ambulated on the first postoperative day and had a mild degree of limping on the first postoperative day, which settled to a normal gait by the third postoperative day. No major complications were noticed in any of the patients, including paresthesia. The postoperative PA pelvis revealed a well-healed anterior iliac crest in all the patients without any crestal deformities, and the growth of the hemi pelvis remained normal in comparison to the opposite side. None of the patients presented with any long-term issues of gait or paresthesia as evaluated 2-4 years postoperatively.

## Discussion

Because the adult and pediatric anterior iliac crests differ so little, harvesting ICBG in pediatric patients is frequently technique-dependent. The pediatric iliac crest consists of a thick cartilaginous apophysis with medial and lateral cortices that fuse at varying distances from the crest [1-3]. These two factors not only reduce the amount of graft available in children, but they also make graft harvesting more sensitive [1].

The cartilaginous apophysis' primary function is to facilitate pelvic growth, and maintaining its vascular integrity is critical to avoiding adverse effects on pelvic growth. This iliac apophysis ossifies between the ages of 14 and 16, at which point the crestal cartilage is replaced with bone. The superior gluteal artery, a branch of the ilio-lumber arteries that courses the lateral surface of the iliac bone, provides the primary vascular supply to the anterior iliac wing, including the overlying apophysis, while the superior iliac circumflex arteries supply the medial surface.

According to Chen et al. [4], removing the periosteum and perichondrium from the lateral and medial surfaces impairs the bilateral blood supply. On the contrary, they proposed that stripping the cortex only on the medial side preserves the lateral vascular supply, thereby improving the healing process. Christodoulou et al. discovered that maintaining the iliac apophysis promotes bone regeneration at the crest, particularly in younger, growing children. As a result, the iliac apophysis must be maintained in good condition.

Various ICBG harvest techniques, such as the clamp shell approach, the lateral-based trap door approach, and the trephine approach, are described in the literature. The clamp-shell approach entails splitting the crest to gain access to the medullary portion. Rossillon et al. found that splitting the apophyseal cartilage preserved natural growth while increasing the thickness of the crest in that region [7].

Though a laterally based trap door approach is used, it requires the stripping of the lateral cortex, which is difficult to do without causing blunt injury to the gluteal musculature due to the thin lateral cortex. Aside from that, lateral muscular compartment injury causes delayed ambulation and is associated with a higher rate of gait disturbances. To address the aforementioned issues, harvesting a medially based trap door is safe and simple, reducing intraoperative and postoperative complications. Long-term studies have shown that in the pediatric age group, replacing the cartilaginous apophysis with its normal anatomical position without disrupting the vascular supply aids in bone stock restoration and can be used as a donor site in the future [8].

The current technique is a modified trap door approach that aims to maintain an intact vascular supply to the cartilaginous cap while preserving the volume of the graft that can be harvested. Repositioning the cartilaginous apophysis not only helps to maintain anatomical integrity but it also helps to restore bone stock at the donor site for future use. The postoperative course following the harvest using this technique revealed minimal postoperative pain, which was managed with topical anesthetics until the third postoperative day, followed by nonsteroidal anti-inflammatory drugs beginning the second postoperative day.

Antalgic gait or limping is the most common gait disturbance observed following graft harvest and has been attributed primarily to dissection of the lateral muscular group or injury or hematoma formation on the medial aspect, which causes inflammation of the iliacus and psoas muscles. However, in our series, early ambulation was possible, and no gait disturbances were observed after the third postoperative day, possibly due to the minimal lateral dissection [9,10].

The lateral cutaneous rami of the ilio hypogastric nerve are susceptible to iatrogenic injury, resulting in temporary loss of sensation; this has been reported to range between 8 and 10% [11,12]. Grossman et al. described a clinical syndrome called meralgia paresthetica, which is characterized by acute, mild pain along the distribution of the aforementioned nerve [13]. This has been attributed to a nerve injury near the abdominal aponeurosis. However, the current technique reduced the incidence, most likely due to layer-wise dissection, as described by Miskowski [14].

The trephine approach is ineffective in children because it destroys the cartilaginous apophysis. It has been demonstrated that injury or excision of more than two-thirds of the apophyseal cartilage retards hemi pelvic growth. As a result, maintaining the integrity of the apophyseal cartilage is critical to avoiding negative consequences [15,16].

A limitation of our study is the small sample size. Further multi-centric studies with larger sample sizes would provide important information about maintaining the integrity of the apophyseal cartilage.

## Conclusions

Preservation of the cartilaginous apophysis during the harvest of ICBG in pediatric patients is essential to maintain the anatomical and functional integrity of the anterior iliac wing. The current technique of cartilage preservation for ICBG is one of the best possible ways to avoid complications and minimize the morbidity associated with the harvest of ICBG in children.
